# Aluminum Nanoparticles Affect Human Platelet Function In Vitro

**DOI:** 10.3390/ijms24032547

**Published:** 2023-01-29

**Authors:** Dominik Taterra, Bendik Skinningsrud, Sigurd Lauritzen, Przemysław A. Pękala, Dawid Szwedowski, Iwona M. Tomaszewska, Krzysztof A. Tomaszewski

**Affiliations:** 1 International Evidence-Based Anatomy Working Group, 30-034 Krakow, Poland; 2 Department of Anatomy, Jagiellonian University Medical College, 31-034 Krakow, Poland; 3 Faculty of Medicine and Health Sciences, Andrzej Frycz Modrzewski Krakow University, 30-705 Krakow, Poland; 4 Department of Organic Chemistry, Faculty of Pharmacy, Collegium Medicum in Bydgoszcz, Nicolaus Copernicus University in Toruń, 85-094 Bydgoszcz, Poland; 5 Department of Medical Education, Jagiellonian University Medical College, 31-034 Krakow, Poland; 6 Scanmed St. Raphael Hospital, 30-693 Krakow, Poland

**Keywords:** aluminum nanoparticles, ceramic prostheses, platelet aggregation

## Abstract

Endoprostheses are prone to tribological wear and biological processes that lead to the release of particles, including aluminum nanoparticles (Al NPs). Those particles can diffuse into circulation. However, the toxic effects of NPs on platelets have not been comprehensively analyzed. The aim of our work was to investigate the impact of Al NPs on human platelet function using a novel quartz crystal microbalance with dissipation (QCM-D) methodology. Moreover, a suite of assays, including light transmission aggregometry, flow cytometry, optical microscopy and transmission electron microscopy, were utilized. All Al NPs caused a significant increase in dissipation (D) and frequency (F), indicating platelet aggregation even at the lowest tested concentration (0.5 µg/mL), except for the largest (80 nm) Al NPs. A size-dependent effect on platelet aggregation was observed for the 5–20 nm NPs and the 30–50 nm NPs, with the larger Al NPs causing smaller increases in D and F; however, this was not observed for the 20–30 nm NPs. In conclusion, our study showed that small (5–50 nm) Al NPs caused platelet aggregation, and larger (80 nm) caused a bridging–penetrating effect in entering platelets, resulting in the formation of heterologous platelet–Al NPs structures. Therefore, physicians should consider monitoring NP serum levels and platelet activation indices in patients with orthopedic implants.

## 1. Introduction

Since its introduction, total joint arthroplasty has become one of the most successful treatment options in orthopedic surgery, effectively restoring patient mobility, relieving pain and improving the patients’ quality of life [1,2,3]. Total hip arthroplasty (THA) and total knee arthroplasty (TKA) are the most prevalent. Each year, more than one million THA procedures are performed globally, and over 100,000 and 700,000 TKA procedures are performed in the UK and in the US, respectively [4,5]. Both procedures are considered primary treatments in cases of advanced osteoarthritis [6], as well as for resections of malignancies and hemophilic arthropathy [7]. There has also been a growing interest in joint arthroplasty in neurosurgery. Total disc replacement (TDR) is becoming an increasingly popular alternative to cervical and lumbar fusion in the treatment of degenerative disc disease [8], offering preserved function of the affected intervertebral joints and favorable long-term outcomes [9].

A variety of bearing materials has been introduced to reduce the rate of revision and complications associated with THA, TKA and TDR implants. While many currently used hip and knee replacements are of the metal-on-polyethylene (MoP) bearing type, metal-on-metal (MoM) bearings have seen a resurgence during the last two decades due to a lower risk of dislocation [10,11,12]. Hip, knee and intervertebral endoprostheses are known to be prone to both tribological wear and biological processes that cause the release of nanoparticles (NPs, particles with a size of <100 nm), metallo-organic complexes, inorganic oxides and free ions [13,14] that can diffuse into the circulation and accumulate in various organs [15]. Local adverse effects of wear debris are well-described in the literature with regard to cytotoxicity, genotoxicity and soft-tissue damage [16,17,18].

As ceramic prostheses offer advantages in terms of low wear and complication rates, they are now becoming increasingly preferred as bearing surfaces and especially desirable for young and physically active patients [19,20]. These include ceramic-on-ceramic (CoC) and ceramic-on-polyethylene (CoP) bearings, and the ceramic components of these bearings are often composed of alumina (Al_2_O_3_) [20].

Ceramic implants containing Al_2_O_3_ are shown to release alumina NPs ranging from 5 to 90 nm [21,22] in size, and toxicological research on implant-derived NPs has largely been centered around those composed of cobalt and chromium (Co-Cr) [17,18,23,24,25]. A wide array of applications using Al_2_O_3_ NP are emerging in nanomedicine [26,27,28], and studies on NPs, such as those found in ceramic implants and nanomedical devices, and their effect on cells are largely limited to lymphocytes, macrophages and fibroblasts [29,30,31,32]. 

Given that NPs such as Al NPs are sufficiently small (<100 nm) to diffuse into the bloodstream, their impact on blood should be a concern. When in the bloodstream, these NPs may interact with various components and may have potential pro-aggregating or anti-aggregating effects on platelets, which could increase the risk of thrombotic or hemorrhagic events in patients with THA, TKA and TDR implants. This is particularly crucial as these patients are at higher risk of postoperative venous thromboembolism after TKA or THA procedures [33]. However, the effects of Al NPs in circulation are not well-understood. Previous studies on other types of nanoparticles (e.g., carbon) have shown that, once in the bloodstream, their presence can lead to widespread thrombosis [34,35]. 

As a consequence, the safety of these NPs and their interactions with blood components such as platelets remain unaddressed. Therefore, our study aimed to analyze the effect of Al_2_O_3_ (Al) NPs on platelet function in vitro utilizing quartz crystal microbalance with dissipation (QCM-D) methodology. Since traditional methods for measuring platelet aggregation may not detect aggregation caused by nanoparticles (NPs) at the concentrations found in vivo, a more sensitive methodology is needed. In this study, QCM-D was used as it is a highly sensitive tool for detecting platelet aggregation and microaggregation [36,37]. QCM-D allows for the real-time examination of platelet microaggregates, which precede the formation of larger platelet thrombi, in a concentration-dependent, flow-dependent, and shear stress-dependent manner that mimics physiological conditions [36].

## 2. Results

### 2.1. Characterization of Aluminium Nanoparticle

The size, polydispersity index (PDI) and zeta potential of each NP are presented in Table 1.

Confirmation of NP characteristics was performed with a Zetasizer^®^ Nano ZS, which were found to be in compliance with the data provided by the manufacturer.

### 2.2. Aluminium Nanoparticles Cause Platelet Aggregation 

**Figure 1:**
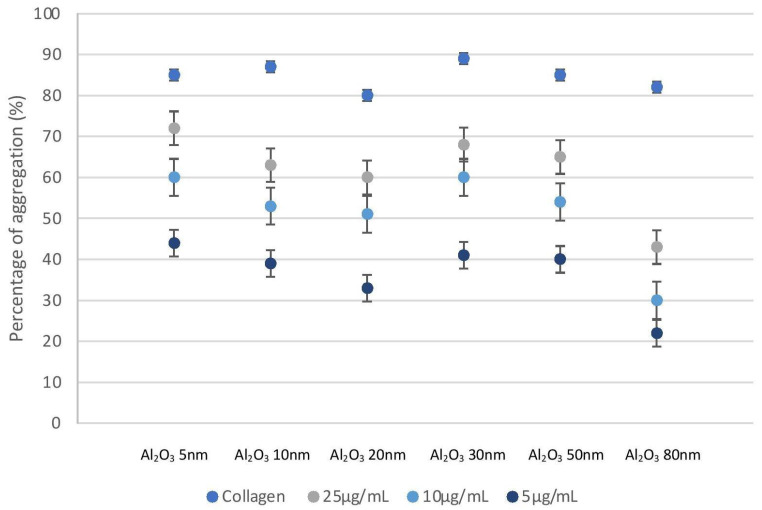
Light transmission aggregometry results showing concentration-dependent platelet aggregation. Collagen (5 µg/mL) was used as positive control. Value of aggregation was given at 10 min of LTA assessment.

Platelet aggregation was measured using light transmission aggregometry (LTA). Platelet aggregation was dependent on concentration and incubation of PRP with the NPs confirmed platelet aggregation at all tested concentrations (25, 10 and 5 µg/mL). Incubation of PRP with lower concentrations of Al NPs showed varied levels of platelet aggregation (Figure 1). Platelets showed the most substantial level of platelet aggregation in the presence of collagen, whereas platelets showed no induction of platelet aggregation in the presence of PPP.

The effects of Al NPs on platelet aggregation were further studied at lower concentrations using QCM-D. Platelet-rich plasma perfusion of the sensor crystals without the application of platelet modulators caused an observable increase in dissipation (D) and a decrease in frequency (F). The platelet deposits on the sensor surface were confirmed using optical microscopy and TEM. Protein-poor plasma served as a control for deposited proteins on the surface of the sensor crystals.

There were significant changes in F and D compared to controls under flow when sensor crystals were perfused with PRP in the presence of Al_2_O_3_ 5 nm, Al_2_O_3_ 10 nm, Al_2_O_3_ 20 nm, Al_2_O_3_ 30 nm, Al_2_O_3_ 50 nm, and Al_2_O_3_ 80 nm (at a concentration of 5, 2.5, 1 and 0.5 µg/mL).

**Figure 2:**
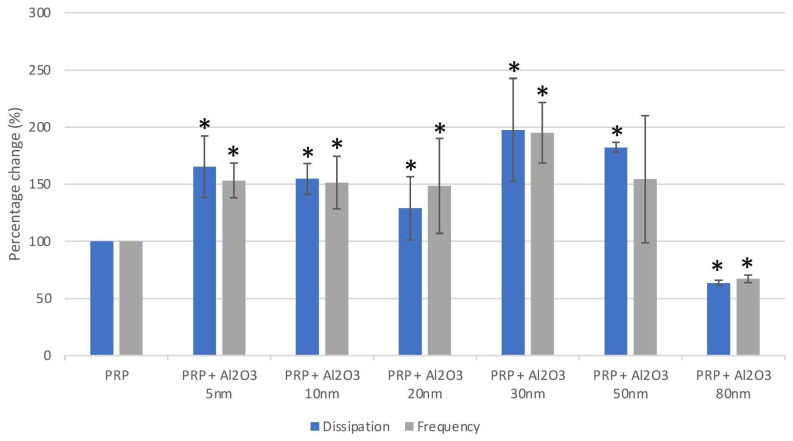
Effects of aluminum nanoparticles (0.5 µg/mL) on platelet-rich plasma (PRP) using quartz crystal microbalance with dissipation. Data are expressed as mean ± standard deviation. * indicates significant difference when compared to control (PRP).

All Al NPs caused a significant increase in D and F, indicating platelet aggregation even at the lowest tested concentration (0.5 µg/mL) as seen in Figure 2, except for the largest (80 nm) Al NPs. The largest increase in D occurred when the platelets were incubated with Al 30 nm with a mean D of 197 ± 45.0. A size-dependent effect on platelet aggregation was observed for the 5–20 nm NPs and the 30–50 nm NPs with larger Al NPs causing smaller increases in D and F; however, this was not observed for the 20–30 nm NPs (Figure 2). Aluminum 80 nm NPs caused statistically significantly smaller changes in D and F compared to all other NP sizes.

### 2.3. Flow Cytometry Shows No Platelet Activation in the Presence of Al Nanoparticles

**Figure 3:**
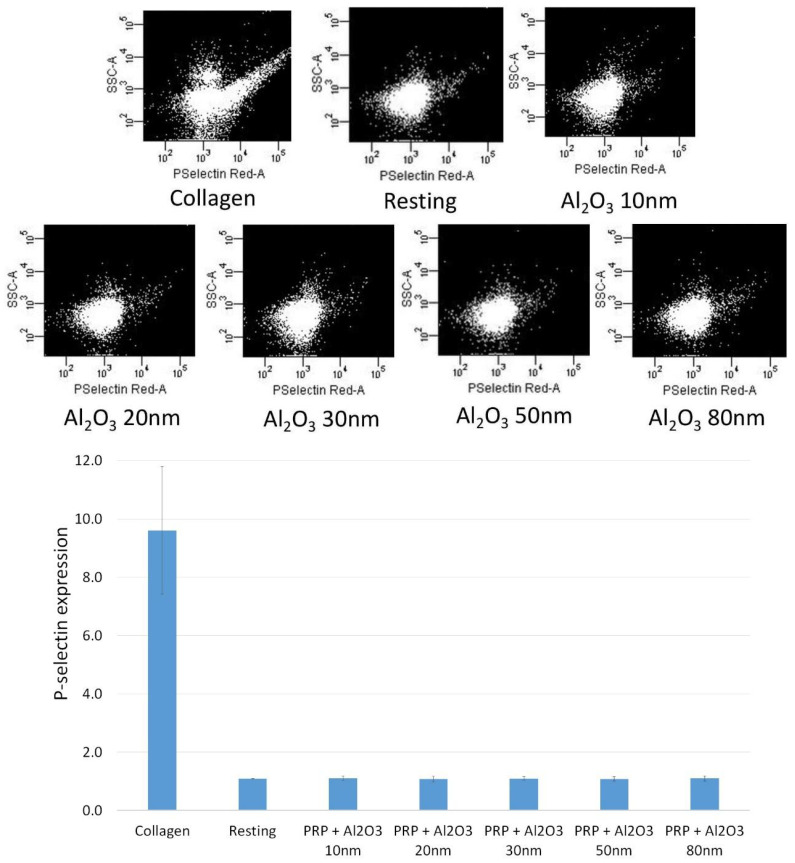
Flow cytometry results showing analyses of P-selectin expression on platelets in the presence of aluminum nanoparticles (5 µg/mL). Collagen (5 µg/mL)-induced aggregation was used as positive control and resting platelets were used as negative control.

The effect of Al_2_O_3_ 5 nm, Al_2_O_3_ 10 nm, Al_2_O_3_ 20 nm, Al_2_O_3_ 30 nm, Al_2_O_3_ 50 nm, and Al_2_O_3_ 80 nm on the level of platelet P-selectin expression in the presence of the tested NPs (concentration of 5 µg/mL) was measured using flow cytometry, and no significant increase in the number of P-selectin copies on their surface was observed (Figure 3).

### 2.4. Optical Microscopy Shows Platelet Aggregates on QCM-D Crystals

**Figure 4:**
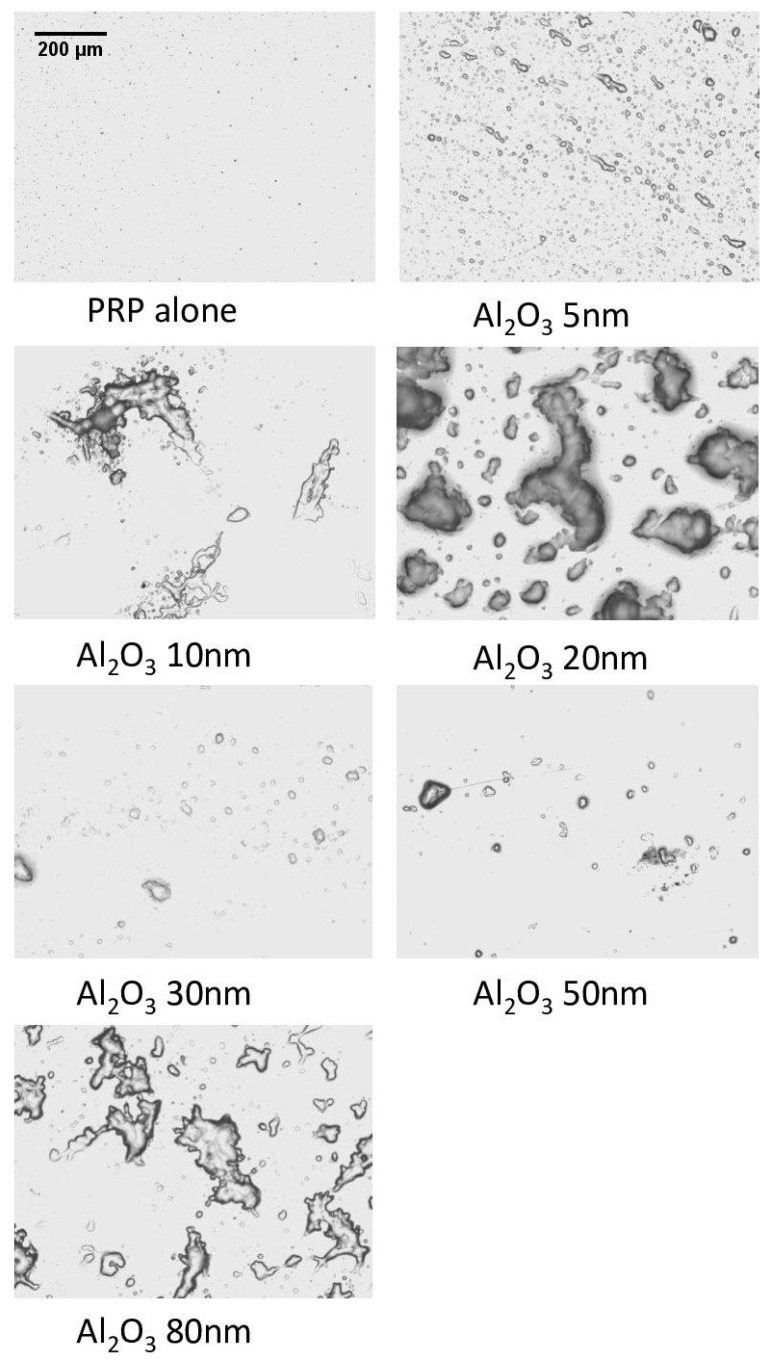
Micrographs of the surface of quartz crystals as viewed through optical microscopy. All light microscopy images are at 20× and at 0.5 ug/mL concentration.

The results obtained when incubating PRP with Al NPs in QCM-D were confirmed utilizing optical microscopy. During this analysis, large platelet aggregates on the sensor crystal surfaces were observed (Figure 4). Contrary to QCMD, the largest NPs (Al 80 nm) showed collections of platelets that resembled aggregates.

### 2.5. Transmission Electron Microscopy Shows Different Mechanisms of Interaction with Platelets for Small (5–50 nm) and Large (80 nm) Al Nanoparticles

**Figure 5:**
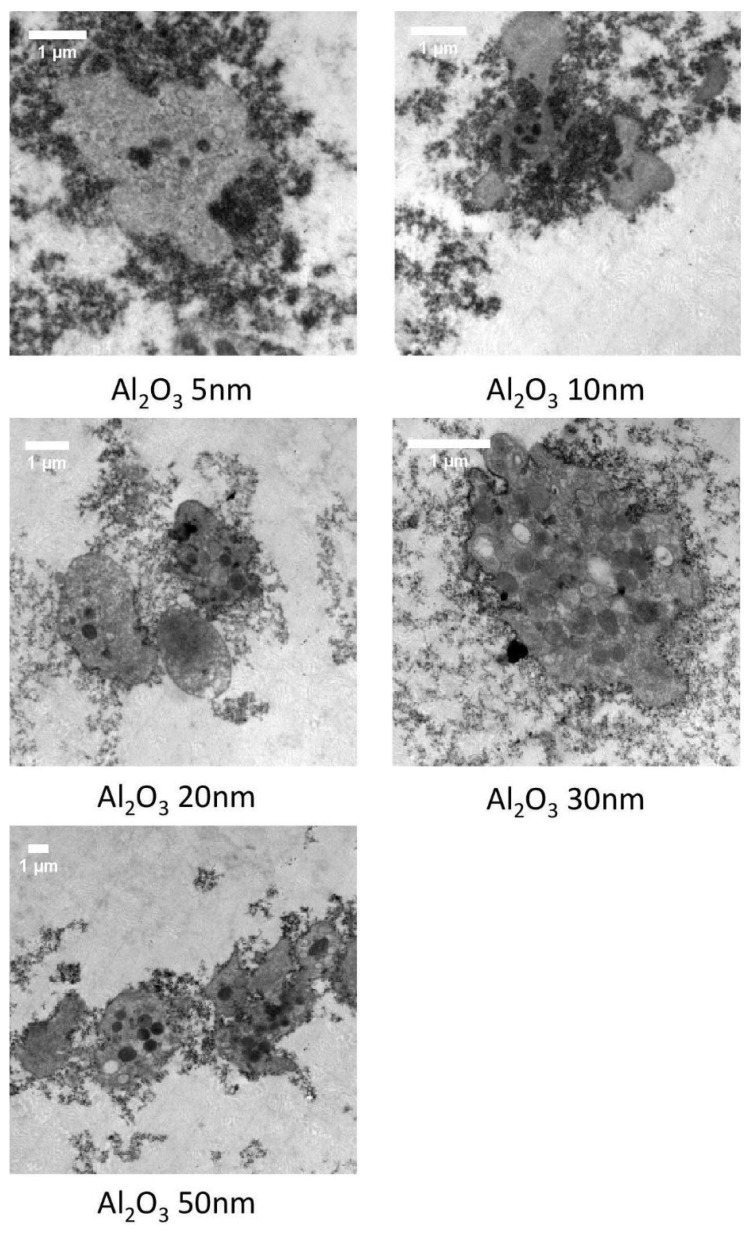
Transmission electron microscopy micrographs of the surface of sensor quartz crystals following perfusion of platelet-rich plasma in the presence of aluminum (5 nm–50 nm) nanoparticles (at a concentration of 0.5 µg/mL) showing platelet activation and aggregation.

**Figure 6:**
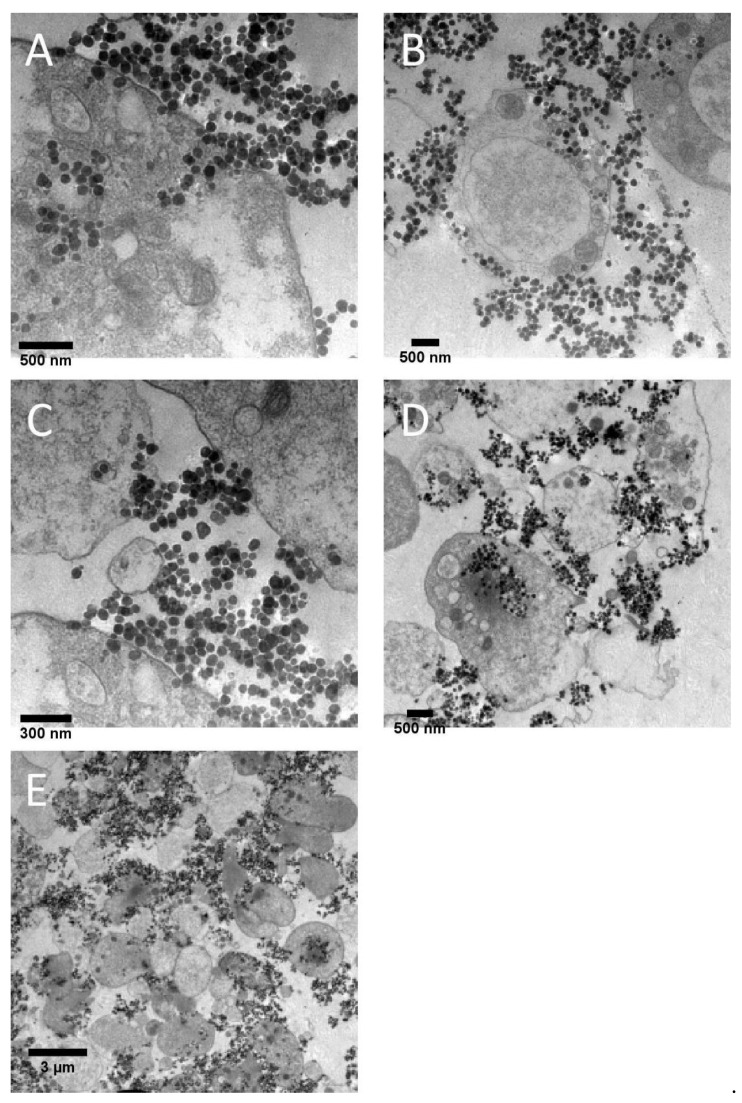
Transmission electron microscopy micrographs of the surface of sensor quartz crystals showing platelet entry (**A**,**B**), bridging (**C**,**D**) and collections of several poorly activated platelets resembling aggregates (**E**) following perfusion of platelet-rich plasma in the presence of aluminum (80 nm) nanoparticles (at a concentration of 0.5 µg/mL).

Platelet-rich plasma samples incubated in the presence of Al NPs were fixed and studied under TEM. The images revealed that Al_2_O_3_ 5 nm–50 nm NPs led to platelet activation and aggregation (Figure 5). The results of TEM analysis also showed that the largest Al NPs (80 nm) did not cause platelet aggregation; however, they entered (Figure 6A,B) and bridged (Figure 6C,D) platelets, resulting in several weakly activated platelets bridged by NPs, resembling aggregates (Figure 6E).

## 3. Discussion

The aim of this study was to analyze the effects of Al NPs on platelet function, including activation and aggregation. We used Al NPs ranging from 5 nm to 80 nm in size because particles of this size are present in wear debris derived from CoC and CoP bearings [21,22,38], which are increasing in popularity due to their advantages in terms of wear when compared to MoM and MoP bearings [20]. To quantify platelet aggregation induced by Al NPs Al, we used a very sensitive QCM-D method that measures flow-induced platelet microaggregation [36,39,40,41], followed by microscopic analysis of the samples.

We found that Al NPs modulated platelet function in a size-dependent manner. Al NPs at concentrations from 5 to 50 nm caused platelet aggregation, while larger Al NPs (80 nm) did not. The Al NPS 80 nm entered and bridged platelets, forming several non-activated (quiescent) platelets resembling platelet aggregates.

Enhanced platelet activation is a known contributor to the pathophysiology of cardiovascular events [42], and as a consequence, the presence of Al NPs in the bloodstream may pose a considerable risk in their development. Several other studies have pointed to the possible adverse cardiovascular effects elicited by NPs in vitro and in vivo when present in the circulation [43,44,45]. This is of particular concern to those suffering from diabetes, malignancies, obstructive pulmonary disease or other conditions where increased platelet activation and hyper-coagulability is observed⁠ [46]. Moreover, Al NPs have been used in biosensors, drug delivery systems and a variety of other applications in nanomedicine [26]. Recently, the use of Al NPs as bactericidal agents against multi-drug resistant bacteria has been of particular interest [47] due to their ability to cross the bacterial cell membrane, triggering the loss of membrane integrity likely through the generation of reactive oxygen species [27,28].

We found that Al NPs have the ability to stimulate flow-dependent platelet microaggregation as shown by QCM-D. In contrast, previous studies examining the effects of Al NPs at 0.2 nm and 50 nm on human fibroblasts reported no alteration of cell behavior [29,30]. There are, however, findings suggesting that harmful effects of Al NPs on fibroblasts may depend on the length of exposure, as shown by reduced cell viability after 2 days in vitro [48].

Interestingly, the cytotoxic potential of Al NPs on macrophages has been shown to be shape-dependent rather than size-dependent [31]. As observed in our study using platelets, Al NPs exert cell-penetrating abilities, which is consistent with previous findings using human fibroblasts [49], emphasizing the importance of understanding mechanisms of NP entry to the cell and possible subsequent intracellular effects. Therefore, more extensive research on how alumina NPs interact with all human cell types is needed, especially for those found in the circulation.

Serum metal ion levels are elevated in patients that have previously undergone THA, TKA and TDR procedures [50], which are reported to correspond to the degree of implant wear in vivo [50,51]. Interestingly, adverse tissue reactions were also reported when serum metal ion levels were found to be within normal ranges [52]. It may be because only a handful of studies consider serum Al ion levels in their analysis. These are predominantly based on small sample sizes of patients that underwent CoC arthroplasty [53,54,55].

The measurement of platelet microaggregation using QCM-D confirmed our LTA results and showed varying levels of platelet aggregation in all investigated types of NPs. Interestingly, while the largest Al NPs (80 nm) did not cause platelet aggregation, as seen in QCM-D, in optical microscopy, these NPs showed platelets in close proximity to each other, resembling platelet aggregates. The results of TEM analysis showed that the largest Al NPs (80 nm) did not cause platelet activation and aggregation. Instead, Al NPs entered platelets, with little or no evidence for platelet activation, while at the same time bridging platelets together, forming an aggregate-like mass. Moreover, pairwise comparisons of platelet aggregation due to Al NPs showed a size-dependent effect on platelet aggregation for the 5–20 nm NPs and the 30–50 nm NPs, with larger Al NPs causing smaller increases in D and F; however, this was not observed for the 20–30 nm NPs. This could be explained by differences in zeta potentials between those NPs, with NPs of higher positive charge potentially having a stronger interaction with negatively charged platelets [56]. Furthermore, the study by Dwivedi et al. [57] showed that NPs of identical composition and surface charge but differing size can exert different and even opposing effects on cell membrane and surfaces. In platelets, these differences in interaction with the surface membrane can affect the way NPs interact with platelet receptors and signaling molecules, ultimately affecting the strength and mechanism of platelet activation and aggregation. 

Research on platelets and their role in the formation of thrombus at the sites of vascular injury has revealed the complex relationship between two distinct systems involved in platelet activation. These include biochemical factors, such as extracellular matrix proteins, and biomechanical factors, such as shear forces. The increased plasma coagulation caused by NPs is usually explained by platelet activation or induction of the intrinsic coagulation pathway [46]. Interestingly, nanoparticle-induced platelet activation is thought to have a variety of possible underlying mechanisms. First, there is evidence for interactions with platelet receptors such as GPIIB/IIIa, which results in a conformational change of this receptor and subsequent platelet activation [58]. Moreover, the bridging of adjacent non-activated platelets by NPs, potentially increasing platelet aggregation, has also been reported in the literature [59]. Other possible mechanisms may include the alteration of intracellular calcium levels, which is one of the key mediators of platelet activation and aggregation [60,61]. Our flow cytometry results did not show a statistically significant difference between the expression of P-selectin among the various NP sizes. However, the size-dependent platelet modulation observed in the presence of Al NPs in our novel QCM-D study may be explained by other mechanisms for platelet activation in vivo. For small Al NPs (5–50 nm), standard platelet aggregation mechanisms could potentially lead to thrombosis. For larger Al NPs (80 nm), the following two mechanisms occur: (1) nanoparticle entry into platelets may result in platelet lysis and the release of intracellular calcium stores; (2) bridging of adjacent non-activated platelets may form aggregate-like masses. 

While this study analyzed NP–platelet interactions under flow conditions, mimicking a physiological state, it did not take into consideration intrinsic relationships between red blood cells, endothelial cells and platelets due to different fluid shear stress, which affect platelet aggregation [62]. Increased fluid shear stress activates platelets through conformational changes of the von Willebrand factor and its binding to platelet’s GPIbα receptor [63]. This can be further mediated by the presence of NPs [62,64]. Moreover, previous research has shown that NPs affect the rheological properties of human blood. Gold or silver NPs increase the blood’s viscosity, while others decrease its viscosity in vitro [65]. A study by Gomez-Garcia et al. [66] found that NPs in circulation accumulate in a model organism in regions of flow disturbances, as observed in pathologic conditions such as atherosclerosis. Taking into consideration that patients often have accompanying cardiovascular disease after THA, TKA and TDR procedures, it could be expected that exposure to Al NPs under favorable conditions could lead to similar or even more robust platelet activation in vivo as compared to what was observed in this study. 

For our study, we selected commercially available, well-characterized Al NPs. However, there is evidence that Al NPs are present in wear debris derived from the most common CoC and CoP bearings [23,67]. Therefore, we suggest that the levels of Al NPs and platelet activation indices should be monitored in patients undergoing THA, TKA and TDR procedures.

## 4. Materials and Methods

### 4.1. Reagents

All reagents were purchased from Sigma-Aldrich (St. Louis, MO, USA) unless otherwise stated.

### 4.2. Dispersion and Characterization of Nanoparticles

All NPs were purchased from US Research Nanomaterials Inc. (Houston, TX, USA). The NPs selected for this study were: Al_2_O_3_ 5 nm (US3007); Al_2_O_3_ 10 nm (US7020); Al_2_O_3_ 20 nm (US3023); Al_2_O_3_ 30 nm (US7030); Al_2_O_3_ 50 nm (US3004); Al_2_O_3_ 80 nm (US3008). 

Aqueous dispersion of the NPs was performed using phosphate-buffered saline (PBS) and platelet-poor plasma (PPP) at a concentration of 1µg/mL in each individual container. A Zetasizer^®^ Nano ZS (Malvern Instruments Ltd., Malvern, UK) and a DTS 1060C clear disposable zeta cell (Malvern Instruments Ltd., Malvern, UK) were used to measure the size and zeta potential for each NP at 25 °C.

### 4.3. Blood Collection and Platelet Isolation

Peripheral blood samples were taken from healthy participants and deposited into a container with a 3.13% (9:1 ratio) sodium citrate solution in order to prevent coagulation. Informed consent was obtained from the participants in written form, and participants were required not to have used any substances that could potentially affect platelet function for at least two weeks preceding the study. Platelet-rich plasma (PRP) and platelet-poor plasma (PPP) were prepared and centrifuged as previously described [68]. Platelet-rich plasma was diluted with PBS until a concentration of 250,000 platelets/μL was achieved [39]. This research was approved by the Trinity College Dublin Faculty of Health Sciences Research Ethics Committee and the Jagiellonian University Medical College Bioethical Committee (approval number 122.6120.184.2016).

### 4.4. Platelet Aggregation Measured by Light Transmission Aggregometry

To test the ability of NPs to affect platelet aggregation, a whole blood Lumi-aggregometer (Chrono-Log Corporation, Havertown, PA, USA) linked with an Aggrolink data reduction system (810DR; Chrono-log) was used [34]. Sonication of NPs was performed for 10 min in a water bath at 37 °C. Incubation of platelet-rich plasma samples with NPs at 25, 10 and 5 µg/mL concentrations was performed, followed by a 10-minute recording of their effects. As a positive control, collagen (Chronolog) (5 µg/mL)-induced platelet aggregation was employed. Platelet-poor plasma served as reference blank. Percentage of maximal aggregation (PPP transmission set at 100%) was used to calculate the data.

### 4.5. Platelet Activation Monitored by Flow Cytometry

To prevent activation of platelets, no stirring or vortexing of the samples was carried out. Flow cytometry was used to measure the level of P-selectin expressed by platelets with an NP concentration of 5 µg/mL. Collagen (5 µg/mL)-induced aggregation served as positive control, whereas resting platelets were used as negative control. After collagen-induced aggregation had reached 50% maximal light transmission as measured by light transmission aggregometry, the samples (PRP) with the NPs present were then incubated for 5 min in a dark environment at room temperature with saturating concentrations (10 μg/mL) of the PAC-1 FITC (Becton Dickinson, Franklin Lakes, NJ, USA) antibody. Following incubation, dilution of the samples was performed using FACSFlow™ (BD Biosciences, Franklin Lakes, NJ, USA) followed by BD FACSCanto™ II (BD Biosciences, Franklin Lakes, NJ, USA) analysis no longer than 5 min after dilution. Single-stained platelet samples were subject to flow cytometry analysis as previously discussed [34]. Identification of the platelet population was performed as specified by their forward- and side-scattered characteristics [69] and analysis of individual platelets for fluorescence was carried out to measure antibody binding followed by correction for autofluorescence and determination of mean fluorescence intensity. Fluorescence analysis was performed for every sample employing a logarithmic scale with at least 30,000 events recorded per sample [39]. Analysis of data was performed using FACSArray software (v 1.0.3; BD Biosciences, Franklin Lakes, NJ, USA) and antibody binding was expressed as the percentage of platelets positive for the antibody (where the values for collagen were considered as 100%).

### 4.6. Platelet Aggregation Measured Using Quartz Crystal Microbalance with Dissipation

To investigate platelet aggregation under flow conditions, a quartz crystal microbalance with dissipation (QCM-D) (Q-Sense™ E4, Biolin Scientific, Stockholm, Sweden) was employed [36,39]. Spin-coating of gold-coated quartz crystals (QSX 301, Biolin Scientific, Stockholm, Sweden, fundamental frequency: 4.95 MHz) with a 0.5% *w*/*v* polystyrene solution was performed utilizing a Polos 150i spin coater (SPS-Europe, Putten, Netherlands). These were incubated in the presence of 100 µg/mL of fibrinogen (dissolved in PBS) for one hour preceding the investigations. Perfusion of the PRP samples (250,000/µL) was performed at 37 °C and a flow rate of 100 µL/minute utilizing a peristaltic pump (Ismatec, IMS 935, Glattburg, Switzerland), both with and without the investigated NPs. Nanoparticles that induced platelet aggregation detectable at a level of 5 µg/mL in light transmission aggregometry or flow cytometry were added to the samples (PRP) and analyzed using QCM-D. To assess the highest level of dilution where the investigated NPs no longer caused platelet aggregation, the NPs were examined at 0.5–5.0 µg/mL. Before the QCM-D aggregation analysis, sonication of the NPs was performed for 10 min. Real-time monitoring of platelet aggregation was then conducted for 30 min using acquisition Q-Sense software (QSoft401, v.2.8), which was recorded as changes in frequency and dissipation. Platelet-poor-plasma served as a control to investigate whether NPs could create changes in frequency and dissipation. Changes in frequency and dissipation were recorded for 30 min with PPP perfused at 100 μL/minute in the presence or absence of investigated NPs.

### 4.7. Optical Microscopy

To microscopically examine aggregate deposits on the sensor surfaces, an Olympus BX43 optical microscope was used. The crystals were then taken for bright field microscopic study using a 20× objective directly after perfusion of QCM-D with NPs at 0.5 ug/mL. The capturing of photomicrographs of these samples was performed using an Olympus XM10 digital camera and Cellsense Dimension software (v. 1.9).

### 4.8. Transmission Electron Microscopy

Platelet-rich plasma samples were collected with the investigated NPs (0.5 µg/mL) from QCM-D crystals. The samples were then fixed in a 0.1 m phosphate buffer (pH 7.4) through an added 2% glutaraldehyde and 2% paraformaldehyde of the same volume, followed by incubation of the sample for 1 h at room temperature [34]. Ultra-thin slices were obtained with a Leica microtome and further treated with uranyl acetate and lead citrate staining using an LKB Ultrastainer (Mount Waverley, Vic, Australia); these were, then, examined in a Zeiss (Oberkochen, Germany) EM900 transmission electron microscope (TEM) at an accelerating voltage of 80 kV.

### 4.9. Statistical Analysis

Chi-squared tests were used for categorical data comparison and QCM-D results were expressed as the frequency shift (Hz) from the baseline (Δf) and the dissipation shift (ΔD) from the baseline (IE-6). A decrease in the oscillating frequency (Δf) as mass deposited on the sensor surface was calculated using the Sauerbrey equation [70]. A loss of energy in the system was determined by the dissipation (ΔD), a dimensionless quantity. These two parameters and their changes could then be used to investigate platelet aggregation using QCM-D. Utilizing mixed ANOVAs, Δf and ΔD were analyzed individually at 60 time points ranging between 30 and 1800 s. If a violation of Mauchly’s assumption of sphericity was observed for any of the analyses conducted, the Greenhouse–Geisser adjustment was applied. All other results were subjected to a mean comparison between two groups utilizing the independent samples t-tests, and a p value < 0.05 was considered statistically significant.

## 5. Conclusions

To the authors’ best knowledge, this is the first study to investigate the interactions between Al NPs and platelets using a novel quartz crystal microbalance with dissipation methodology capable of detecting microaggregation. Our study showed significant pro-aggregating effects of Al nanoparticles on platelets even at the lowest tested concentration of 0.5 µg/mL. Small (5–50 nm) Al NPs caused platelet aggregation, and larger Al NPs (80 nm) caused a bridging–penetrating effect in entering platelets, resulting in the formation of heterologous platelet–Al NPs structures. Considering the results of this study, monitoring Al NP levels and platelet function in patients with orthopedic implants should be advised.

## Figures and Tables

**Table 1:** Characteristics of included nanoparticles (Zetasizer).

Nanoparticles	Zeta Size (SD), nm		Polydispersity Index (SD)		Zeta Potential, mV	
	PPP	DDH_2_0	PPP	DDH_2_0	PPP	DDH_2_0
Al_2_O_3_ 5 nm	239	680 (83.3)	0.538	0.587 (0.069)	-	62.6
Al_2_O_3_ 10 nm	42.8	93.1 (0.98)	0.320	0.219 (0.014)	-	46.0
Al_2_O_3_ 20 nm	9.51	39.1 (0.74)	0.425	0.462 (0.052)	-	16.8
Al_2_O_3_ 30 nm	43.9	89.8 (0.26)	0.447	0.245 (0.006)	-	47.7
Al_2_O_3_ 50 nm	113	152 (1.01)	0.141	0.172 (0.027)	-	50.5
Al_2_O_3_ 80 nm	614	1158 (156.00)	0.543	0.606 (0.132)	-	1.46

PPP: platelet-poor plasma, DDH20: double-distilled water.

## Data Availability

The data presented in this study are not publicly available due to legal restrictions.

## References

[1] Canovas F., Dagneaux L. (2018). Quality of life after total knee arthroplasty. Orthop. Traumatol. Surg. Res..

[2] Learmonth I.D., Young C., Rorabeck C. (2007). The operation of the century: Total hip replacement. Lancet.

[3] Patel A., Pavlou G., Mújica-Mota R.E., Toms A.D. (2015). The epidemiology of revision total knee and hip arthroplasty in England and Wales: A comparative analysis with projections for the United States. a study using the national joint registry dataset. Bone Jt. J..

[4] Ferguson R.J., Palmer A.J., Taylor A., Porter M.L., Malchau H., Glyn-Jones S. (2018). Hip replacement. Lancet.

[5] Kremers H.M., Larson D.R., Crowson C.S., Kremers W.K., Washington R.E., Steiner C.A., Jiranek W.A., Berry D.J. (2014). Prevalence of total hip and knee replacement in the United States. J. Bone Jt. Surg. Am. Vol..

[6] Wood A.M., Brock T.M., Heil K., Holmes R., Weusten A. (2013). A Review on the Management of Hip and Knee Osteoarthritis. Int. J. Chronic Dis..

[7] Solimeno L.P., Pasta G. (2017). Knee and Ankle Arthroplasty in Hemophilia. J. Clin. Med..

[8] Ament J.D., Yang Z., Nunley P., Stone M.B., Kim K.D. (2014). Cost-effectiveness of cervical total disc replacement vs fusion for the treatment of 2-level symptomatic degenerative disc disease. JAMA Surg..

[9] Othman Y.A., Verma R., Qureshi S.A. (2019). Artificial disc replacement in spine surgery. Ann. Transl. Med..

[10] Xia Z., Ricciardi B.F., Liu Z., von Ruhland C., Ward M., Lord A., Hughes L., Goldring S.R., Purdue E., Murray D. (2017). Nano-analyses of wear particles from metal-on-metal and non-metal-on-metal dual modular neck hip arthroplasty. Nanomed. Nanotechnol. Biol. Med..

[11] Sands D., Schemitsch E.H. (2017). The Role of Metal-on-Metal Bearings in Total Hip Arthroplasty and Hip Resurfacing. HSS J..

[12] Bozic K.J., Kurtz S., Lau E., Ong K., Chiu V., Vail T.P., Rubash H.E., Berry D.J. (2009). The epidemiology of bearing surface usage in total hip arthroplasty in the United States. J. Bone Jt. Surg. Ser. A.

[13] Matusiewicz H. (2014). Potential release of in vivo trace metals from metallic medical implants in the human body: From ions to nanoparticles—A systematic analytical review. Acta Biomater..

[14] Papageorgiou I., Brown C., Schins R., Singh S., Newson R., Davis S., Fisher J., Ingham E., Case C.P. (2007). The effect of nano- and micron-sized particles of cobalt-chromium alloy on human fibroblasts in vitro. Biomaterials.

[15] Urban R.M., Jacobs J.J., Tomlinson M.J., Gavrilovic J., Black J., Peoc’h M. (2000). Dissemination of wear particles to the liver, spleen, and abdominal lymph nodes of patients with hip or knee replacement. J. Bone Jt. Surg. Ser. A.

[16] Drummond J., Tran P., Fary C. (2015). Metal-on-Metal Hip Arthroplasty: A Review of Adverse Reactions and Patient Management. J. Funct. Biomater..

[17] Onega T., Baron J., MacKenzie T. (2006). Cancer after total joint arthroplasty: A meta-analysis. Cancer Epidemiol. Biomark. Prev..

[18] Gill H.S., Grammatopoulos G., Adshead S., Tsialogiannis E., Tsiridis E. (2012). Molecular and immune toxicity of CoCr nanoparticles in MoM hip arthroplasty. Trends Mol. Med..

[19] Jeffers J.R.T., Walter W.L. (2012). Ceramic-on-ceramic bearings in hip arthroplasty: State of the art and the future. J. Bone Jt. Surg. Ser. B.

[20] Hu C.Y., Yoon T.R. (2018). Recent updates for biomaterials used in total hip arthroplasty. Biomater. Res..

[21] Zhang Y.F., Zheng Y.F., Qin L. (2011). A comprehensive biological evaluation of ceramic nanoparticles as wear debris. Nanomed. Nanotechnol. Biol. Med..

[22] Wooley P.H. (2014). How Has the Introduction of New Bearing Surfaces Altered the Biological Reactions to Byproducts of Wear and Modularity?. Clin. Orthop. Relat. Res..

[23] Bijukumar D.R., Segu A., Souza J.C.M., Li X.J., Barba M., Mercuri L.G., Jacobs J.J., Mathew M.T. (2018). Systemic and local toxicity of metal debris released from hip prostheses: A review of experimental approaches. Nanomed. Nanotechnol. Biol. Med..

[24] Behl B., Papageorgiou I., Brown C., Hall R., Tipper J.L., Fisher J., Ingham E. (2013). Biological effects of cobalt-chromium nanoparticles and ions on dural fibroblasts and dural epithelial cells. Biomaterials.

[25] Taterra D., Skinningsrud B., Pękala P.A., Tomaszewska I.M., Marycz K., Radomski M.W., Tomaszewski K.A. (2021). In vitro effects of cobalt and chromium nanoparticles on human platelet function. Nanotoxicology.

[26] Hassanpour P., Panahi Y., Ebrahimi-Kalan A., Akbarzadeh A., Davaran S., Nasibova A.N., Khalilov R., Kavetskyy T. (2018). Biomedical applications of aluminium oxide nanoparticles. Micro Nano Lett..

[27] Wang L., Hu C., Shao L. (2017). The antimicrobial activity of nanoparticles: Present situation and prospects for the future. Int. J. Nanomed..

[28] Ansari M.A., Khan H.M., Alzohairy M.A., Jalal M., Ali S.G., Pal R., Musarrat J. (2015). Green synthesis of Al_2_O_3_ nanoparticles and their bactericidal potential against clinical isolates of multi-drug resistant Pseudomonas aeruginosa. World J. Microbiol. Biotechnol..

[29] Faye P.A., Roualdes O., Rossignol F., Hartmann D.J., Desmoulière A. (2017). Engulfment of ceramic particles by fibroblasts does not alter cell behavior. Biomed. Mater..

[30] Tsaousi A., Jones E., Case C.P. (2010). The in vitro genotoxicity of orthopaedic ceramic (Al_2_O_3_) and metal (CoCr alloy) particles. Mutat. Res. Genet. Toxicol. Environ. Mutagen..

[31] Yamamoto A., Honma R., Sumita M., Hanawa T. (2004). Cytotoxicity evaluation of ceramic particles of different sizes and shapes. J. Biomed. Mater. Res. Part A.

[32] Catelas I., Petit A., Marchand R., Zukor D.J., Yahia L., Huk O.L. (1999). Cytotoxicity and macrophage cytokine release induced by ceramic and polyethylene particles in vitro. J. Bone Jt. Surg. Ser. B.

[33] Pedersen A.B., Mehnert F., Sorensen H.T., Emmeluth C., Overgaard S., Johnsen S.P. (2014). The risk of venous thromboembolism, myocardial infarction, stroke, major bleeding and death in patients undergoing total hip and knee replacement: A 15-year retrospective cohort study of routine clinical practice. Bone Joint J..

[34] Radomski A., Jurasz P., Alonso-Escolano D., Drews M., Morandi M., Malinski T., Radomski M.W. (2005). Nanoparticle-induced platelet aggregation and vascular thrombosis. Br. J. Pharmacol..

[35] Mills N.L., Törnqvist H., Gonzalez M.C., Vink E., Robinson S.D., Söderberg S., Boon N.A., Donaldson K., Sandström T., Blomberg A. (2007). Ischemic and Thrombotic Effects of Dilute Diesel-Exhaust Inhalation in Men with Coronary Heart Disease. N. Engl. J. Med..

[36] Santos-Martinez M.J., Tomaszewski K.A., Medina C., Bazou D., Gilmer J.F., Radomski M.W. (2015). Pharmacological characterization of nanoparticle-induced platelet microaggregation using quartz crystal microbalance with dissipation: Comparison with light aggregometry. Int. J. Nanomed..

[37] Larkin C.M., Breen E.P., Tomaszewski K.A., Eisele S., Radomski M.W., Ryan T.A., Santos-Martinez M.-J. (2018). Platelet microaggregation in sepsis examined by quartz crystal microbalance with dissipation technology. Platelets.

[38] Mochida Y., Boehler M., Salzer M., Bauer T.W. (2001). Debris from failed ceramic-on-ceramic and ceramic-on-polyethylene hip prostheses. Clin. Orthop. Relat. Res..

[39] Santos-Martinez M.J., Inkielewicz-Stepniak I., Medina C., Rahme K., D’Arcy D.M., Fox D., Holmes J.D., Zhang H., Radomski M.W. (2012). The use of quartz crystal microbalance with dissipation (QCM-D) for studying nanoparticle-induced platelet aggregation. Int. J. Nanomed..

[40] Tonda-Turo C., Carmagnola I., Ciardelli G. (2018). Quartz crystal microbalance with dissipation monitoring: A powerful method to predict the in vivo behavior of bioengineered surfaces. Front. Bioeng. Biotechnol..

[41] Fatisson J., Azari F., Tufenkji N. (2011). Real-time QCM-D monitoring of cellular responses to different cytomorphic agents. Biosens. Bioelectron..

[42] Badimon L., Padró T., Vilahur G. (2012). Atherosclerosis, platelets and thrombosis in acute ischaemic heart disease. Eur. Heart J. Acute Cardiovasc. Care.

[43] Abukabda A.B., Stapleton P.A., Nurkiewicz T.R. (2016). Metal Nanomaterial Toxicity Variations within the Vascular System. Curr. Environ. Health Rep..

[44] Feng Q., Liu Y., Huang J., Chen K., Huang J., Xiao K. (2018). Uptake, distribution, clearance, and toxicity of iron oxide nanoparticles with different sizes and coatings. Sci. Rep..

[45] Cao Y., Gong Y., Liao W., Luo Y., Wu C., Wang M., Yang Q. (2018). A review of cardiovascular toxicity of TiO_2_, ZnO and Ag nanoparticles (NPs). BioMetals.

[46] Fröhlich E. (2016). Action of Nanoparticles on Platelet Activation and Plasmatic Coagulation. Curr. Med. Chem..

[47] Baptista P.V., McCusker M.P., Carvalho A., Ferreira D.A., Mohan N.M., Martins M., Fernandes A.R. (2018). Nano-strategies to fight multidrug resistant bacteria—“A Battle of the Titans”. Front. Microbiol..

[48] Germain M.A., Hatton A., Williams S., Matthews J.B., Stone M.H., Fisher J., Ingham E. (2003). Comparison of the cytotoxicity of clinically relevant cobalt-chromium and alumina ceramic wear particles in vitro. Biomaterials.

[49] Radziun E., Dudkiewicz Wilczyńska J., Ksiazek I., Nowak K., Anuszewska E.L., Kunicki A., Olszyna A., Zabkowski T. (2011). Assessment of the cytotoxicity of aluminium oxide nanoparticles on selected mammalian cells. Toxicol. Vitr..

[50] Hannemann F., Hartmann A., Schmitt J., Lützner J., Seidler A., Campbell P., Delaunay C.P., Drexler H., Ettema H.B., García-Cimbrelo E. (2013). European multidisciplinary consensus statement on the use and monitoring of metal-on-metal bearings for total hip replacement and hip resurfacing. Orthop. Traumatol. Surg. Res..

[51] Reito A., Berryman F., Young S., Pandit H.G., Lainiala O., Eskelinen A., McConnell J., Judge A., Matharu G.S., Murray D.W. (2017). Blood Metal Ion Thresholds to Identify Patients with Metal-on-Metal Hip Implants at Risk of Adverse Reactions to Metal Debris. J. Bone Jt. Surg..

[52] Grammatopoulos G., Munemoto M., Pollalis A., Athanasou N.A. (2017). Correlation of serum metal ion levels with pathological changes of ARMD in failed metal-on-metal-hip-resurfacing arthroplasties. Arch. Orthop. Trauma Surg..

[53] Weissinger M., Grübl A., Pöll G. (2011). Serum-cobalt levels with metal-on-metal bearings in the cement-free total hip arthroplasty results covering two years; prospective study. Acta Chir. Orthop. Traumatol. Cech..

[54] Savarino L., Padovani G., Ferretti M., Greco M., Cenni E., Perrone G., Greco F., Baldini N., Giunti A. (2008). Serum ion levels after ceramic-on-ceramic and metal-on-metal total hip arthroplasty: 8-Year minimum follow-up. J. Orthop. Res..

[55] Grübl A., Weissinger M., Brodner W., Gleiss A., Giurea A., Gruber M., Pöll G., Meisinger V., Gottsauner-Wolf F., Kotz R. (2006). Serum aluminium and cobalt levels after ceramic-on-ceramic and metal-on-metal total hip replacement. J. Bone Jt. Surg. Ser. B.

[56] Montague S.J., Patel P., Martin E.M., Slater A., Quintanilla L.G., Perrella G., Kardeby C., Nagy M., Mezzano D., Mendes P.M. (2021). Platelet activation by charged ligands and nanoparticles: Platelet glycoprotein receptors as pattern recognition receptors. Platelets.

[57] Dwivedi M.V., Harishchandra R.K., Koshkina O., Maskos M., Galla H.-J. (2014). Size Influences the Effect of Hydrophobic Nanoparticles on Lung Surfactant Model Systems. Biophys. J..

[58] Tomaszewski K.A., Radomski M.W., Santos-Martinez M.J. (2015). Nanodiagnostics, nanopharmacology and nanotoxicology of platelet-vessel wall interactions. Nanomedicine.

[59] Smyth E., Solomon A., Vydyanath A., Luther P.K., Pitchford S., Tetley T.D., Emerson M. (2015). Induction and enhancement of platelet aggregation in vitro and in vivo by model polystyrene nanoparticles. Nanotoxicology.

[60] Van Kruchten R., Braun A., Feijge M.A.H., Kuijpers M.J.E., Rivera-Galdos R., Kraft P., Stoll G., Kleinschnitz C., Bevers E.M., Nieswandt B. (2012). Antithrombotic potential of blockers of store-operated calcium channels in platelets. Arterioscler. Thromb. Vasc. Biol..

[61] Jun E.A., Lim K.M., Kim K., Bae O.N., Noh J.Y., Chung K.H., Chung J.H. (2011). Silver nanoparticles enhance thrombus formation through increased platelet aggregation and procoagulant activity. Nanotoxicology.

[62] Saikia J., Mohammadpour R., Yazdimamaghani M., Northrup H., Hlady V., Ghandehari H. (2018). Silica Nanoparticle–Endothelial Interaction: Uptake and Effect on Platelet Adhesion under Flow Conditions. ACS Appl. Bio Mater..

[63] Rana A., Westein E., Niego B., Hagemeyer C.E. (2019). Shear-Dependent Platelet Aggregation: Mechanisms and Therapeutic Opportunities. Front. Cardiovasc. Med..

[64] Chen Y.Y., Syed A.M., MacMillan P., Rocheleau J.V., Chan W.C.W. (2020). Flow Rate Affects Nanoparticle Uptake into Endothelial Cells. Adv. Mater..

[65] Michalczuk U., PRzekop R., Moskal A. (2022). The effect of selected nanoparticles on rheological properties of human blood. Bull. Polish Acad. Sci. Tech. Sci..

[66] Gomez-Garcia M.J., Doiron A.L., Steele R.R.M., Labouta H.I., Vafadar B., Shepherd R.D., Gates I.D., Cramb D.T., Childs S.J., Rinker K.D. (2018). Nanoparticle localization in blood vessels: Dependence on fluid shear stress, flow disturbances, and flow-induced changes in endothelial physiology. Nanoscale.

[67] Urban R.M., Tomlinson M.J., Hall D.J., Jacobs J.J. (2004). Accumulation in liver and spleen of metal particles generated at nonbearing surfaces in hip arthroplasty. J. Arthroplast..

[68] Radomski M., Moncada S. (1983). An improved method for washing of human platelets with prostacyclin. Thromb. Res..

[69] Shattil S.J., Cunningham M., Hoxie J.A. (1987). Detection of activated platelets in whole blood using activation-dependent monoclonal antibodies and flow cytometry. Blood.

[70] Sauerbrey G. (1959). Verwendung von Schwingquarzen zur Wägung dünner Schichten und zur Mikrowägung. Z. Für Phys..

